# Fundamental Investigation into Tool Wear and Surface Quality in High-Speed Machining of Ti6Al4V Alloy

**DOI:** 10.3390/ma14237128

**Published:** 2021-11-23

**Authors:** Adel T. Abbas, Essam A. Al Bahkali, Saeed M. Alqahtani, Elshaimaa Abdelnasser, Noha Naeim, Ahmed Elkaseer

**Affiliations:** 1 Department of Mechanical Engineering, College of Engineering, King Saud University, P.O. Box 800, Riyadh 11421, Saudi Arabia; ebahkali@ksu.edu.sa (E.A.A.B.); 439106256@student.ksu.edu.sa (S.M.A.); 2 Department of Production Engineering and Mechanical Design, Port Said University, Port Fuad 42526, Egypt; Alshymaa.gamal@eng.psu.edu.eg (E.A.); noha.fouaad@eng.psu.edu.eg (N.N.); ahmed.elkaseer@kit.edu (A.E.); 3 Institute for Automation and Applied Informatics, Karlsruhe Institute of Technology, 76344 Karlsruhe, Germany

**Keywords:** high-speed machining, Ti6Al4V, flank wear, crater wear, carbide insert, surface roughness

## Abstract

This paper reports a fundamental investigation consisting of systematic trials into the response of Ti6Al4V alloy to high-speed machining using carbide inserts. It is a useful extension to work previously published, and aims at assessing the impact of the process parameters, depth of cut, cutting speed and feed rate in addition to cutting length, and their interrelations, on observed crater and flank wear and roughness of the machined surface. The results showed that abrasion was the most important flank wear mechanism at high speed. It also showed that increased cutting length accelerated crater wear more than exhibited flank wear and had considerable effect on surface roughness. In particular, crater wear increased by over 150% (on average), and flank wear increased by 40% (on average) when increasing cutting length from 40 to 120 mm. However, cutting the same length increased surface roughness by 50%, which helps explain how progression of tool wear leads to deteriorated surface quality. ANOVA was used to perform statistical analyses of the measured data and revealed that cutting length and depth of cut had the greatest effect on both crater and flank wear of the cutting tool. These results confirm that high-speed machining of Ti6Al4V alloy is a reliable process, with cutting speed identified as having a relatively small influence on the tool wear and resultant roughness of the machined surface relative to other parameters.

## 1. Introduction

High-speed machining has been attracting considerable interest as it offers higher productivity and lower manufacturing costs with acceptable surface quality [1]. A number of studies have noted the advantages of high-speed machining, including lower cutting force required and less vibration [2], decreased cross sectional area of chips due to higher cutting speeds (v_c_) [3], and better chip evacuation [4]. Thus, it has definite benefits when machining difficult-to-cut materials. Richardson et al. [5] carried out high-speed tests machining aerospace aluminum alloy and reported achieving higher feed rates and cutting speeds, with lower cutting temperatures. Hamdan et al. [6] carried out milling tests on stainless steel alloy using high-speed machining and found increasing cutting speed reduced both surface roughness (Ra) and resultant cutting force. Wang et al. [4] has reported similar results for Inconel 718 due to the transition from a ductile to a brittle regime. However, high-speed machining has drawbacks, including higher strain rates and working temperatures, which can affect surface quality and tool life [7]. Twardowski et al. [8] reported that tool life is mainly dependent on the geometries and coating material of carbide inserts when hardened steel is machined at high speed. Niknam et al. [9] studied the machining responses of titanium metal matrix alloy using CBN inserts at high speed and found that different wear mechanisms were detected, including adhesion, abrasion, and oxidation, with the ability to govern tool wear and obtainable surface roughness albeit with different combinations of process parameters. Thus, the success of high-speed machining is subject to accurately specifying the appropriate ranges of cutting speed and feed rate for the material to be worked to avoid negative effects [10].

Titanium alloys have a wide range of applications in industries including biomedical, marine, chemical, automotive, and aerospace [11]. Thus, their machining with high efficiency is a very important issue for industry [12]. However, titanium alloys are superalloys and machining them can be problematic, especially at high speeds. This has resulted in many recommendations to machine at low values of cutting speed [10]. These alloys tend to possess relatively low thermal conductivity with consequent higher cutting temperatures, they also tend to chemically react with cutting tool materials and can weld to them [13], both of these can be major factors impeding their machinability and adversely affecting tool wear, tool life, and surface quality [14]. Nevertheless, due to economic and technical requirements, high-speed machining is in increasing demand by industry where it is essential to maintain high surface integrity of components while rapidly removing large quantities of material [7].

The high-speed machining of titanium alloys has been investigated by numerous research teams. Abdelnasser et al. [15] carried out a study comparing high-speed and conventional hard turning of the most widely used titanium alloy (Ti6Al4V) using polycrystalline diamond (PCD) inserts. The metal removal rate (MRR) was doubled, and there was smoother machining with a more than 10% reduction in surface roughness when using high-speed rather than conventional machining. The authors attributed this to the increased temperature that occurs when using high-speed machining, which causes thermal softening of the material and reduces material strength near the cutting zone. Nor did the authors report any side effects, such as increased tool wear, due to the use of high-speed machining.

Accelerated tool wear that occurs during the machining of titanium alloys as a result of elevated temperatures is an important issue that impacts on their machinability and increases manufacturing costs [16]. There have been numerous investigations into the effect of various cutting tool inserts [17,18] and cutting conditions [19] on the life of tools used to machine titanium-based alloys. Sharma et al. [20] investigated the machining of Ti6Al4V specimens using tungsten carbide inserts without coatings for cutting speed between 20 and a high cutting speed of 1100 m·min^−1^. The results indicated a decrease in cutting force at speeds between 500 and 1100 m·min^−1^ due to an increase in workpiece temperature sufficient to soften the material. However, at the maximum cutting speed it was noted that the temperature became very unstable with rapid wearing of the tool and build-up of edge formation. An examination of flank wear (V_B_) showed adhesion wear at speeds between 20 and 250 m·min^−1^. Between speeds of 500 and 800 m·min^−1^ the authors observed adhesion-diffusion-dissolution wear, and at 1100 m·min^−1^ found indications of attrition wear. Da Silva et al. [21] used different types of cutting inserts when high-speed machining Ti6Al4V and found for cutting speed between 240 and 300 m·min^−1^, PCD inserts gave much better wear than cemented carbide inserts. The latter gave acceptable results only in the range 60–120 m·min^−1^. Kaya et al. [22] claimed that when machining Ti6Al4V with PCD inserts, the inserts did not work well at low cutting speed, 70 m·min^−1^, but surface roughness and tool wear were reduced when machining at the higher cutting speed of 130 m·min^−1^. Inserts other than PCD, such as polycrystalline cubic boron nitride (PCBN) also referred to as CBN, gave superb results when high-speed machining titanium alloys [9,23]. These materials can retain their hardness at the high temperatures associated with high-speed machining [14,24]. However, PCD and PCBN have lower fracture toughness than carbides and using them can increase machining costs [25].

Studies have investigated ways to reduce the temperature when machining titanium alloys to help reduce tool wear and surface roughness. Qin et al. [26] highlighted the importance of the combination of tool coating material and cooling strategy that has to be identified to extend tool life when turning titanium alloy. Tascioglu et al. [27] compared different cooling systems when machining titanium alloy using carbide inserts and reported that high-pressure coolant performance was better than the flood condition, followed by minimum quantity lubrication and, finally, machining dry. However, other reported results concluded that a negative effect of high-pressure coolant is it can cause notch wear [28]. Furthermore, Pervaiz et al. [29] found that turning of Ti6Al4V alloy with a flood cooling condition resulted in rougher surfaces at higher feed rates compared to surfaces obtained under dry machining. However, the authors concluded that using MQL (internal) helps in decreasing surface roughness, tool wear, and cutting forces compared to cases of flood and dry machining. Abbas et al. [30] studied machining of Ti6Al4V at high-speeds for different cutting speeds, feed rates, depth of cut (a_p_), and cutting lengths (l) using a fusion approach (multi-objective optimization based on ratio analysis, MOORA) combined with regression analysis and particle swarm algorithms to minimize surface roughness and maximize MMR. Follow-up research by Abbas et al. [31] reported another optimization technique (fuzzy-TOPSIS), to optimize cutting performance by minimizing power consumed, flank wear (V_B_), and surface roughness while simultaneously maximizing MMR. However, although Abbas et al. [30,31] achieved their goal over a wide range of machining conditions, they did not provide an adequate explanation of their results.

This has motivated the authors of this investigation to attempt to bridge this gap and address this omission because as revealed by the literature search, high-speed machining of titanium alloys remains problematic and more studies are needed to optimize both cutting tool selection and cutting conditions to minimize tool wear while maintaining good surface quality, without increasing manufacturing costs. Thus, this paper aims to extend the investigation reported in [30,31] to understand how the various machining factors and their interactions affect surface roughness, flank wear, and crater wear (K_B_) when high-speed machining titanium alloys, in particular Ti6Al4V.

## 2. Experimental Work

The experiments, as described and explained in [30,31], were conducted on 40 mm diameter rods of Ti6Al4V alloy. The mechanical properties and chemical composition of Ti6Al4V are reported in [30]. A CNC turning machine, Emco Concept Turn 45, with a Sinumeric 840-D digital NC system was used. Turning operations were carried out under flood conditions using a cooling pump (2.2 kW). The SVJCL2020K16 (Sandvik Coromant, Stockholm, Sweden) tool holder and VBMT160404-VBMT331-PM carbide inserts (Sandvik Coromant, Stockholm,, Sweden) dwere used [30,31]. The turning tests were conducted on Ti6Al4V for different values of feed rate, depth of cut, cutting speed, and cutting length (see Table 1, modified from [30,31]). The design of the experiments was full factorial.

The cutting performance was assessed in terms of flank wear, crater wear, and surface roughness (Ra). Here it is worth repeating that this paper is an extension for a fundamental investigation of the experimental work of Abbas et al. [30,31] without conducting further experiments. Surface roughness (Ra) values were measured using a Tesa-Rugosurf-90G Roughness and Profile Measurement Gauge. A digital optical microscope (ASKANIA Mikroskop Technik Rathenow GmbH, Rathenow, Switzerland) was used to measure crater and flank face wear on the tool’s rake face.

## 3. Results and Discussion

**Figure 1:**
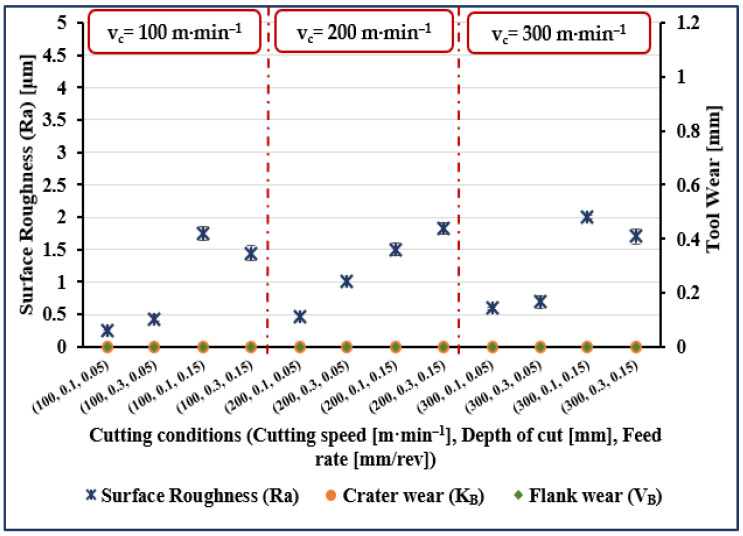
Measured surface roughness (Ra), flank wear (V_B_), and crater wear (K_B_) for trials at cutting length (l) of 5 mm.

**Figure 2:**
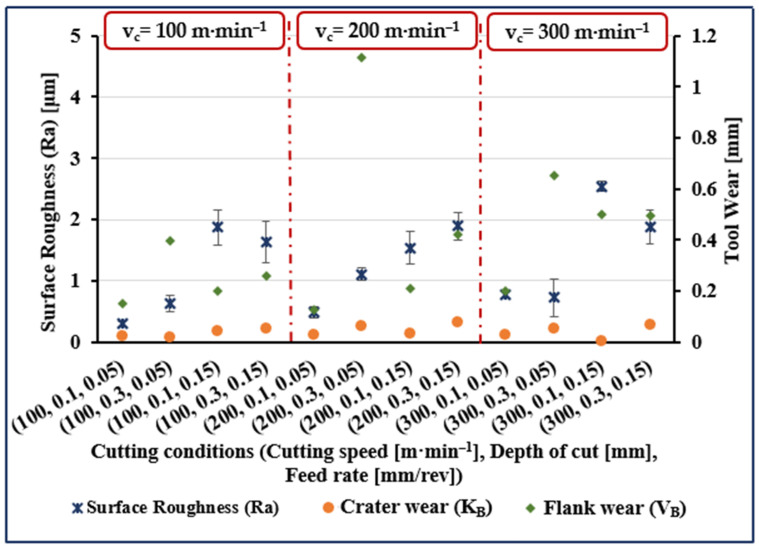
Measured surface roughness (Ra), flank wear (V_B_), and crater wear (K_B_) for trials at cutting length (l) of 40 mm.

**Figure 3:**
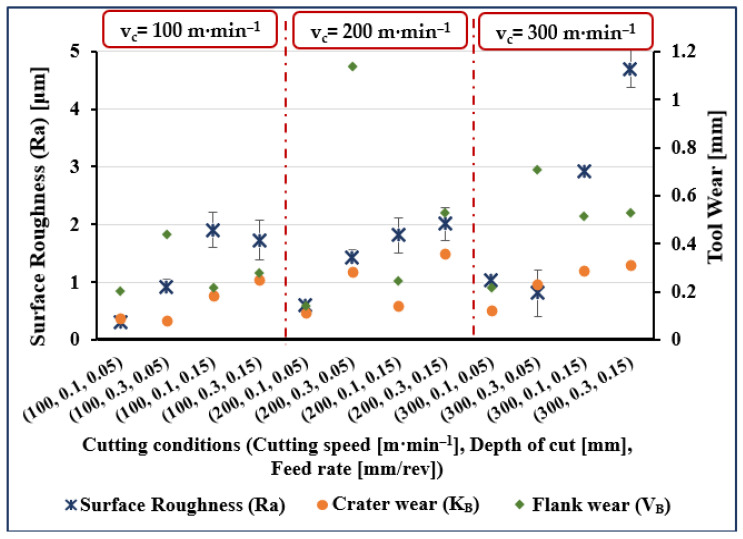
Measured surface roughness (Ra), flank wear (V_B_), and crater wear (K_B_) for trials at cutting length (l) of 80 mm.

**Figure 4:**
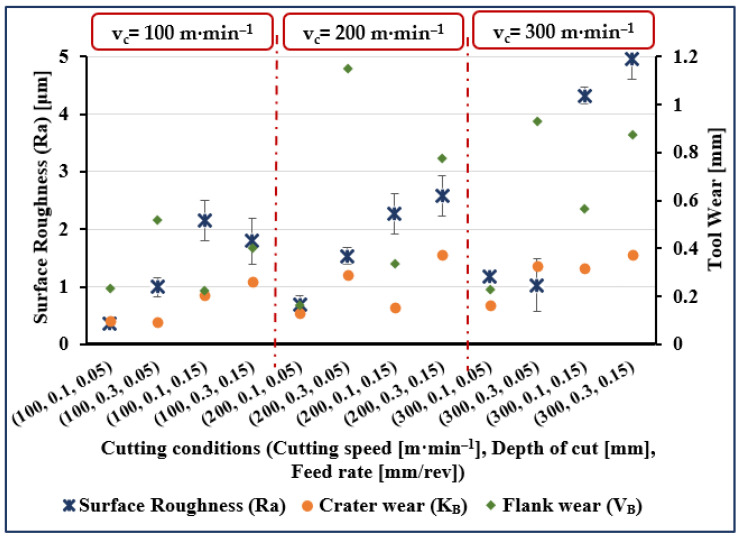
Measured surface roughness (Ra), flank wear (V_B_), and crater wear (K_B_) for trials at cutting length (l) of 120 mm.

The experimental results are presented in Figure 1, Figure 2, Figure 3 and Figure 4. The measured data for surface roughness (Ra), crater wear (K_B_), and flank wear (V_B_) for given values of cutting speed, depth of cut and feed rate, and for cutting length values of 5, 40, 80, and 120 mm are illustrated in Figure 1, Figure 2, Figure 3 and Figure 4, respectively.

Considering surface roughness (Ra), as presented in Figure 1, Figure 2, Figure 3 and Figure 4, a considerable variation can be observed. There is an increase of almost 250% in surface roughness when feed rate increased from 0.05 to 0.15 mm/rev, while all other factors remaining constant. Surface roughness increased by almost 45% when cutting speed increased from 100 to 200 m·min^−1^ and all other parameters remained constant, though a further increase in cutting speed from 200 to 300 m·min^−1^ brought an increase in surface roughness of only 34%. Surface roughness increased by 72% when cutting length increased from 5 to 120 mm, while an increase of 50% when cutting length increased from 40 to 120 mm and an increase of 45% was noted when depth of cut was increased from 0.1 to 0.3 mm.

Measured crater wear and flank wear were zero when cutting length was 5 mm for all values of the other process parameters (see Figure 1). At all other values of cutting lengths, both flank wear and crater wear, were measurable quantities but with flank wear invariably more than crater wear (see Figure 2, Figure 3 and Figure 4). Comparing the results for different values of cutting length, we see crater wear increased by over 150% (on average), and flank wear increased by 40% (on average) when increasing cutting length from 40 to 120 mm. Obviously, cutting length has a greater effect on crater wear than flank wear.

In Figure 2, Figure 3 and Figure 4, on average, crater wear is greater for cutting speed = 200 m·min^−1^ than for cutting speed = 100 m·min^−1^ or 300 m·min^−1^. The average increase in crater wear was 77% when increasing the cutting speed from 100 to 200 m·min^−1^. However, only a slight increase in crater wear (9% on average) was noted when cutting speed was increased from 200 to 300 m·min^−1^. Increasing depth of cut increased crater wear, an increase from 0.1 to 0.3 mm increased crater wear by, on average, 146%.

Figure 2, Figure 3 and Figure 4 show that flank wear increased by 58%, on average, when cutting speed increased from 100 to 200 m·min^−1^. A further increase in cutting speed from 200 to 300 m·min^−1^ increased flank wear by, on average, 33%. A substantial increase in average flank wear of 208% was noted when depth of cut was increased from 0.1 to 0.3 mm. Comparing results, it was seen that crater wear increased by 74% and flank wear by 23% (on average) when feed rate increased from 0.05 to 0.15 mm/rev.

However, certain cases appear to diverge from the general considerations. For instance, the greatest flank wear occurred at the minimum feed rate of 0.05 mm/rev, a moderate cutting speed of 200 m·min^−1^ and a relatively deep depth of cut, 0.3 mm. This confirms the need to further investigate the interactions between cutting parameters and the responses obtained.

### 3.1. Tool Wear in High-Speed Machining of Ti6Al4V

#### 3.1.1. Effects of Cutting Length (l)

**Figure 5:**
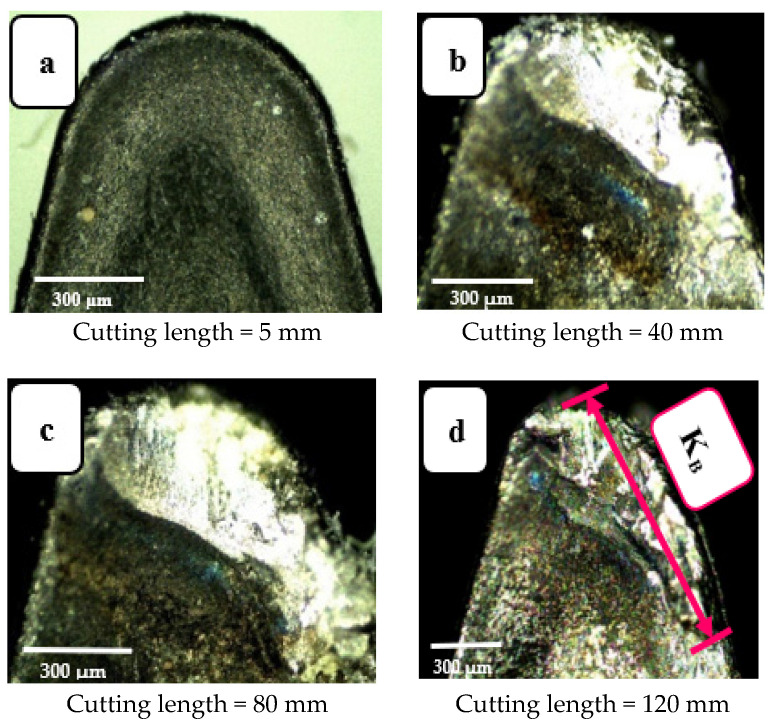
Crater wear images for four cutting lengths: (**a**) l = 5 mm, (**b**) l = 40 mm, (**c**) l = 80 mm, and (**d**) l =120 mm. Cutting speed 200 m·min^−1^; depth of cut 0.3 mm; feed rate 0.15 mm/rev (modified from [30]).

In real machining, the tool continuously interacts with the workpiece throughout the contact length, gradually wearing the tool away in different ways, each to a different degree. Figure 5 shows four images of a worn tool, taken at four different values cutting length: 5, 40, 80, and 120 mm. It is observable that at the start of the cut (i.e., 5 mm, Figure 5a) the tool is fresh with no wear. However, when cutting length is increased to 40 mm, crater wear on the rake surface is clearly visible. When cutting is continued further using the same tool, the crater wear region became deeper and wider (see Figure 5b,c). Finally, at cutting length = 120 mm, the surface profile of the tool rake was quite irregular and showed prominent wear (Figure 5d). This agrees with the results reported in [17].

**Figure 6:**
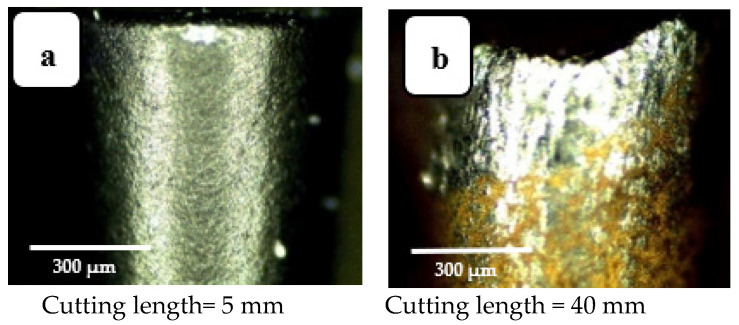

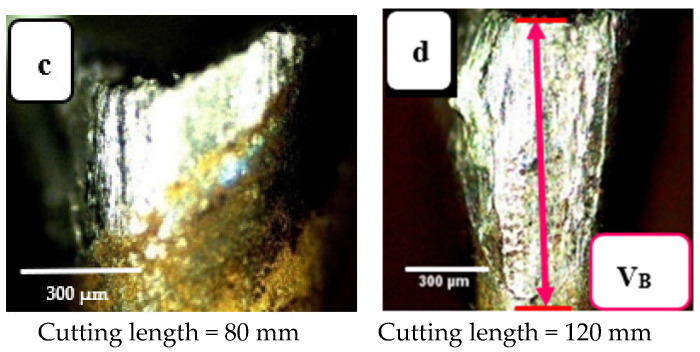
Flank wear images for four cutting lengths: (**a**) l = 5 mm, (**b**) l = 40 mm, (**c**) l = 80 mm, and (**d**) l = 120 mm. Cutting speed 200 m·min^−1^; depth of cut 0.3 mm; feed rate 0.15 mm/rev (modified from [30]).

This wear pattern is credited to continuous chip-rubbing over the tool’s surface. This interaction is one source of heat generation when cutting, and largely accounts for the increased rake surface temperature. Ultimately this leads to the crater wear shown in the images presented in Figure 5. Similarly, Figure 6 shows the increasing flank wear with increase in cutting length. This agrees with the results reported in [14,27]. At cutting length = 120 mm, the depth of the wear band is greatest (see Figure 6d). This is associated with friction between tool and worked surface that is present as long as the process continues, causing the observed abrasion of the turning tool’s outer layer, revealing its inner core.

#### 3.1.2. Effects of Cutting Speed (v_c_)

**Figure 7:**
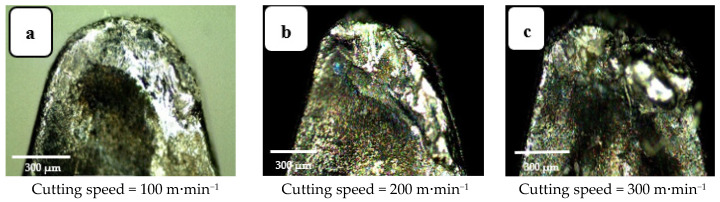
Crater wear images at three cutting speeds: (**a**) v_c_ = 100 m·min^−1^, (**b**) v_c_ = 200 m·min^−1^, and (**c**) v_c_ = 300 m·min^−1^. Depth of cut 0.3 mm, cutting length 120 mm, and feed rate = 0.15 mm/rev.

**Figure 8:**
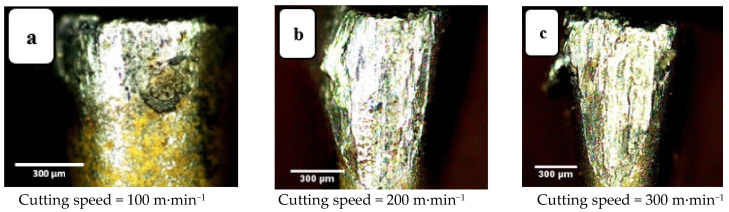
Flank wear images at three cutting speeds: (**a**) v_c_ = 100 m·min^−1^, (**b**) v_c_ = 200 m·min^−1^, and (**c**) v_c_ = 300 m·min^−1^. Depth of cut 0.3 mm, cutting length 120 mm, and feed rate = 0.15 mm/rev.

Machining was performed for three values of cutting speed, 100, 200, and 300 m·min^−1^, the corresponding values of crater wear and flank wear for the tool are presented in Figure 7 and Figure 8, respectively, all other parameters are kept constant. Increasing cutting speed increases wear because an increase in cutting speed requires greater expenditure of mechanical energy, which generates more heat. Most of this heat energy is absorbed by the tool and chips, but while the chips are discarded the tool continues cutting, increasing the thermal energy it absorbs, increasing its temperature and causing crater wear of the tool. This agrees with the results reported in [20,21]. Flank wear, however, is caused by the friction between the tool and workpiece, an abrasion phenomenon that depletes the external layer of the tool insert. Away from the tool tip wear marks are less obvious which indicates that it is the tool tip and regions close to the tip that interact most with the workpiece.

#### 3.1.3. Effects of Feed Rate

**Figure 9:**
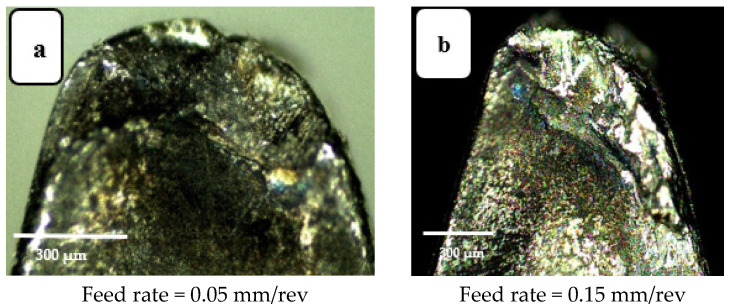
Crater wear images for (**a**) feed rate 0.05 mm/rev and (**b**) feed rate 0.15 mm/rev. Cutting speed 200 m·min^−1^, depth of cut 0.3 mm, cutting length 120 mm (modified from [30]).

**Figure 10:**
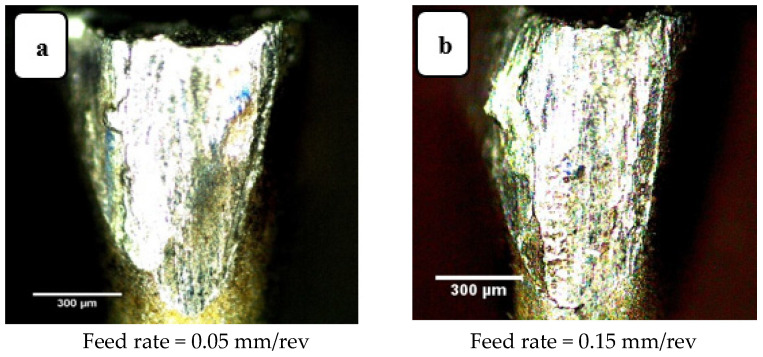
Flank wear images for (**a**) feed rate 0.05 mm/rev and (**b**) feed rate 0.15 mm/rev. Cutting speed 200 m·min^−1^, depth of cut 0.3 mm, cutting length 120 mm (modified from [30]).

Increasing the feed rate increases the interface contact area per unit time, between workpiece and tool. Crater wear and flank wear are shown in Figure 9 and Figure 10, respectively, each for two feed rates. The tool insert “peels” a layer from the workpiece due to the frictional forces between the tool-chip and tool-workpiece. This peeling is different for different zones of the tool. For instance, the area closer to the tool tip is seen to be more susceptible to tool wear than other areas. When the feed rate was increased from 0.05 to 0.15 mm/rev, abrasion also increased, as seen in Figure 9 and Figure 10, which show the irregularities (wear marks) in the worn zone as being deeper and broader for the higher feed rate.

#### 3.1.4. Effects of Depth of Cut (a_p_)

**Figure 11:**
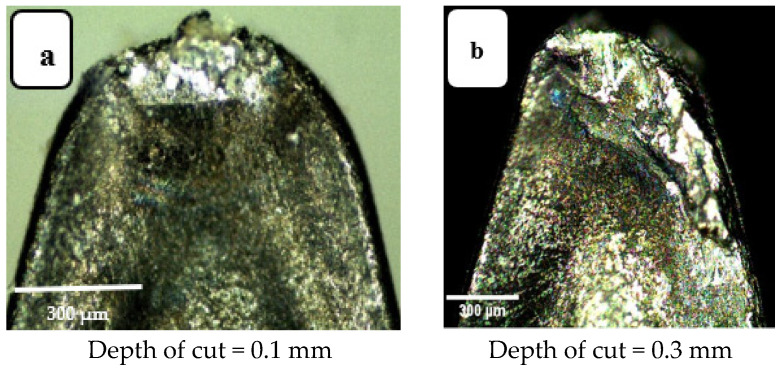
Crater wear images: (**a**) a_p_ = 0.1 mm and (**b**) a_p_ = 0.3 mm. Cutting speed 200 m·min^−1^, cutting length 120 mm, feed rate 0.15 mm/rev, (modified from [30]).

**Figure 12:**
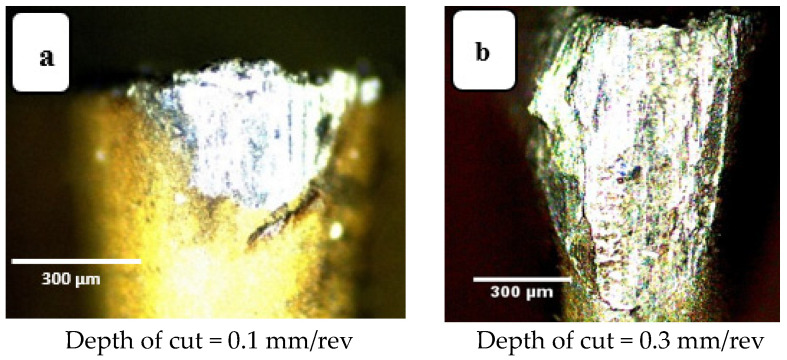
Flank wear images: (**a**) a_p_ = 0.1 mm and (**b**) a_p_ = 0.3 mm_._ Cutting speed 200 m·min^−1^, cutting length 120 mm, feed rate 0.15 mm/rev, (modified from [30]).

Increasing depth of cut also increases the area of active contact between tool and workpiece and, for that reason increasing the depth of cut increases wear. For instance, in Figure 11a, the crater wear at depth of cut of 0.1 mm is only at the tool tip, while that for a larger value of depth of cut, of 0.3 mm, the crater depth covers a larger zone (see Figure 11b). This greater wear can be ascribed to the greater volume of material removal due to a deeper cut. The more material removed, the greater the total cutting force required, the more thermal energy generated, and the greater the tool wear. Looking at flank wear in Figure 12, a similar trend is seen, for larger values of depth of cut, the deeper and wider the wear zone, the greater the flank wear. The active friction area extends further along the flank extending away from the tip of the tool. Again, tool wear is abrasion induced. This agrees with the results reported in [32].

### 3.2. Statistical Analysis

Here, functional correlations are developed to characterize the relationships between machining inputs and responses. Statistical regression using a quadratic expression of the form shown in Equation (1), has been developed using MATLAB,

(1) $$y=b_{0}+\sum^{\;}b_{i}x_{i}+\sum^{\;}b_{\mathrm{ii}}x_{\mathrm{ii}}^{2}+\sum^{\;}b_{\mathrm{ij}}x_{i}x_{j},$$

where y is the response (e.g., K_B_, V_B_, and Ra), b_0_, b_i_, etc., are the regression coefficients to be extracted from the experimental data, and x_i_ and x_j_ are the ith and jth values, respectively, of the input parameter, x (e.g., v_c_, l, a_p_, and feed rate).

The mathematical equations developed for the three responses in terms of cutting speed (v_c_), cutting length (l), depth of cut (a_p_), and feed rate, are presented in Equations (2)–(4). Equation (2) shows the predicted value of crater wear. For this equation, R-squared was 0.872, and adjusted R-squared was 0.829. Equation (3) represents the predicted value of flank wear. Here R-squared was 0.814, and adjusted R-squared was 0.751. Equation (4) shows the predicted value of average surface roughness (Ra). Here R-squared was 0.860, and adjusted R-squared was 0.812.

In the three equations, a_pn_, l_n_, v_cn_, and f_n_ are the values of a_p_, l, v_c_, and feed rate, normalized to lie between (–1, 1).

(2) $$K_{B}=0.21546+0.034917{\;f}_{n}+0.03525{\;v}_{\mathrm{cn}}+0.0380{\;a}_{\mathrm{pn}}+0.10798{\;l}_{n}-0.0060{\;f}_{n}.v_{\mathrm{cn}}\;\;+0.0026667{\;f}_{n}.a_{\mathrm{pn}}+0.013562{\;v}_{\mathrm{cn}}.a_{\mathrm{pn}}+0.023428{\;f}_{n}.l_{n}\;\;+0.031583{\;v}_{\mathrm{cn}}.l_{n-}0.0190{\;v}_{\mathrm{cn}}^{2}-0.086504{\;l}_{n}^{2}\;$$

(3) $$V_{B}=0.48767-0.023917{\;f}_{n}+0.090656{\;v}_{\mathrm{cn}}+0.14413{\;a}_{\mathrm{pn}}+0.24581{\;l}_{n}\;\;+0.027781{\;f}_{n}.v_{\mathrm{cn}}-0.07975{\;f}_{n}.a_{\mathrm{pn}}+0.028594{\;v}_{\mathrm{cn}}.a_{\mathrm{pn}}\;\;-0.0016563{\;f}_{n}.l_{n}+0.068931{\;v}_{\mathrm{cn}}.l_{n}+0.11023{\;a}_{\mathrm{pn}}.l_{n}\;\;-0.086844{\;v}_{\mathrm{cn}}^{2}-0.16319{\;l}_{n}^{2}\;$$

(4) $$R_{a}=1.3685+0.75679{\;f}_{n}+0.41822{\;v}_{\mathrm{cn}}+0.1215{\;a}_{\mathrm{pn}}+0.44288{\;l}_{n}\;\;+0.25334{\;f}_{n}.v_{\mathrm{cn}}-0.056375{\;f}_{n}.a_{\mathrm{pn}}+0.012719{\;v}_{\mathrm{cn}}.a_{\mathrm{pn}}\;\;+0.23807{\;f}_{n}.l_{n}+0.34598{\;v}_{\mathrm{cn}}.l_{n}+0.083135{\;a}_{\mathrm{pn}}.l_{n}\;\;-0.15291{\;v}_{\mathrm{cn}}^{2}-0.092489{\;l}_{n}^{2}\;$$

These three equations represent the regression models and were used to quantify the influence of the four input process parameters (and their mutual interactions) on the measured values of crater wear (K_B_), flank wear (V_B_), and surface roughness (Ra). These results were assessed using one-way ANOVA with 95% confidence level, and presented below is an assessment of those parameters with the most effect on crater wear, flank wear, and surface roughness (Ra).

#### 3.2.1. Tool Wear for High-Speed Machining of Ti6Al4V

**Figure 13:**
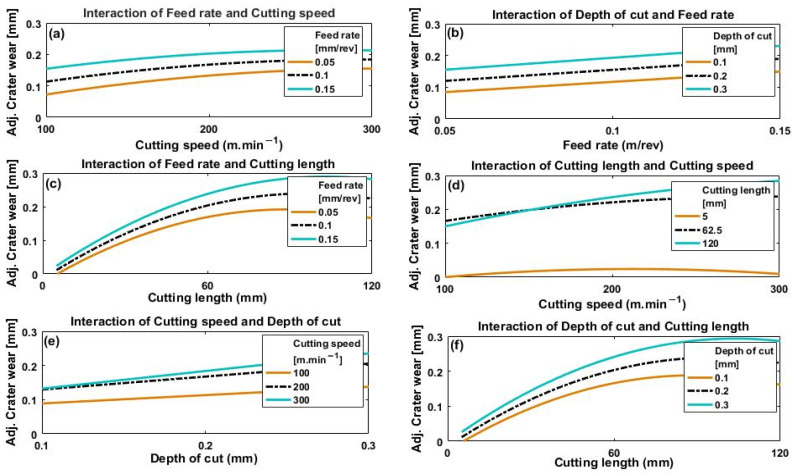
Plots depicting how crater wear is affected by (**a**) cutting speed at different feed rates, (**b**) feed rate for different depths of cut, (**c**) cutting length at different feed rates, (**d**) cutting speed for different cutting lengths, (**e**) depth of cut for different cutting speeds, and (**f**) cutting length for different depths of cut.

**Figure 14:**
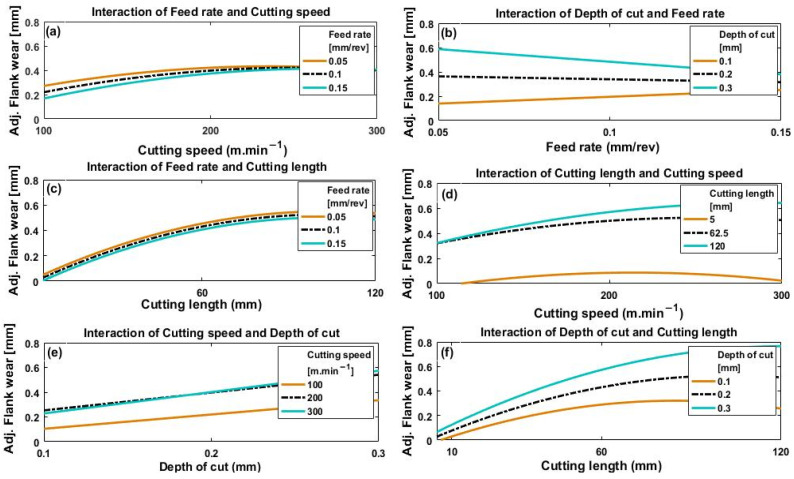
Plots depicting how flank wear is affected by (**a**) cutting speed for different feed rates, (**b**) feed rate for different depths of cut, (**c**) cutting length for different feed rates, (**d**) cutting speed for different cutting lengths, (**e**) depth of cut for different cutting speeds and (**f**) cutting length for different depths of cuts.

Figure 13a–f and Figure 14a–f depict the effect of the interactions of the four process parameters on crater wear and flank wear, respectively.

In Figure 13a and Figure 14a, it can be seen at that for all feed rates, both crater wear and flank wear increased with cutting speed, a trend which levelled off when cutting speed reached between 200 and 300 m·min^−1^. The initial increase in crater wear and flank wear with cutting speed is because of material build-up on the cutting edge of the rake face and increased friction on the flank face of the tool [33]. However, generally, the values of both crater wear and flank wear appear to level off with increase in cutting speed from 200 to 300 m·min^−1^. This is because the increased temperature, due to the faster cutting speed, thermally softened the material of the workpiece [34] with subsequently easier chip removal [32]. The relatively shorter time the workpiece is machined because of a faster cutting speed may also play a role because the time during which workpiece and tool are in contact is reduced, thus less heat will be generated with less tool wear.

Figure 13b shows an approximately linear increase in crater wear with feed rate for the three values of depth of cut used. This effect is caused by greater strain due to a faster feed rate, which raises the cutting temperature and increases crater wear. Figure 13b also shows an increase in crater wear with depth of cut, but there is no clear indication of any significant interaction between depth of cut and feed rate on crater wear (i.e., the three curves remain parallel).

However, it can be seen from Figure 14b that the flank wear response to changes in feed rate and depth of cut showed quite different results. Flank wear increased proportionally to feed rate for a low value of depth of cut (0.1 mm), but was inversely proportional to feed rate at a deeper cut (0.3 mm). The differences between the flank wear values are large at small feed rates but clearly decrease as feed rate increases, and at speeds above about 250 m·min^−1^ the differences are not significant. A possible cause could be the minimum chip thickness effect. At the lowest feed rates, no proper chipping mechanism takes place until the thickness of the chip reaches a certain minimum value after which cutting becomes the dominant mechanism, [35,36], so flank wear tends to increase at low feed rates. These observations need further investigation and will be considered in future work. Comparison of results presented in Figure 13b and Figure 14b show that interaction between depth of cut and feed rate has a greater effect on flank wear than on crater wear.

Figure 13c and Figure 14c show that both flank wear and crater wear increased with increase in cutting length for every feed rate used. This is because the longer the tool and workpiece are in contact, the longer frictional forces are acting. Figure 13c shows that feed rate has a greater effect on crater wear at larger values of cutting length than shorter lengths. No significant interaction was noted between feed rate and cutting length for flank wear (Figure 14c).

Figure 13d and Figure 14d show similar trends in both crater wear and flank wear with cutting speed and cutting length. The longest cutting length (120 mm) had the greatest effect on both crater wear and flank wear, with reduction of the effect at shorter cutting lengths. Additionally, wear at higher values of cutting speed showed greater variation with cutting length than at lower values.

Figure 13e and Figure 14e show increase in crater wear and flank wear with depth of cut for all three values of cutting speed. At greater depths of cut, thicker chips of workpiece material are removed, generating larger stresses in the tool, which accelerates wear. Depth of cut generated greater crater wear and flank wear at higher cutting speed than at lower, though the effect on flank wear was less than on crater wear.

Figure 13f and Figure 14f show that both crater wear and flank wear increased with cutting length and depth of cut, with depth of cut having a more significant effect on wear at longer cutting lengths, and vice versa.

**Figure 15:**
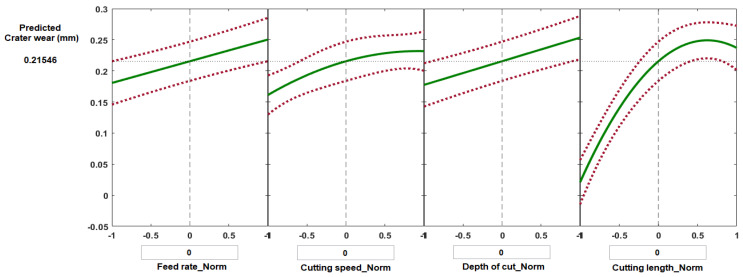
Prediction slice plots created using the developed process regression model for crater wear of Ti6Al4V specimens machined under high-speed mode.

**Figure 16:**
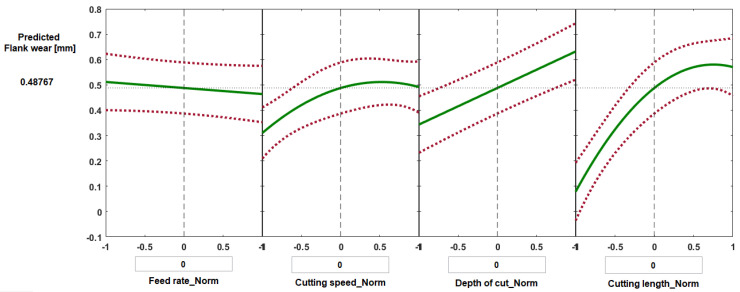
Prediction slice plots created using the developed process regression model for flank wear of Ti6Al4V specimens machined under high-speed mode.

Figure 15 and Figure 16 show selected prediction plots developed using MATLAB regression models to show the particular effects of individual process parameters when all the others were kept constant. The prediction plots can also be used to forecast the values of crater wear and flank wear likely to be obtained with variations of the four process parameters, and thus it should be possible to find the optimal solution to minimize wear. Figure 15 presents the predicted values of crater wear as a function of normalized feed rate, normalized cutting speed, normalized depth of cut, and normalized cutting length. For each plot, the vertical dashed line is when all the process parameters are maintained at their average values: feed rate 0.1 mm/rev, cutting speed 200 m·min^−1^, depth of cut 0.2 mm, and cutting length 62.5 mm, which gave a predicted value of crater wear of 0.215 mm and flank wear of 0.487 mm (Figure 15 and Figure 16, respectively). The red dashed lines are the 95% confidence limits of the predicted responses.

Some experiments were randomly selected to validate the results of regression models for the predicting of crater wear and flank wear (Table 2 and Table 3). The discrepancy between the model predicted values of crater wear and measured results were determined to be less than 8.2%, while the deference was found in the range of 3.3–11% in the case of flank wear for the selected experiments.

ANOVA showed that cutting length has the largest impact on both crater wear and flank wear (*p*-value = 5.9 × 10^−13^ for crater wear and *p*-value = 3.1 × 10^−9^ for flank wear). This was followed by depth of cut (*p*-value = 8.2 × 10^−6^ for crater wear and 4.4 × 10^−7^ for flank wear). Feed rate was the third most significant parameter for crater wear (*p*-value = 3.0 × 10^−5^) with cutting speed last (*p*-value = 3.5 × 10^−4^). With flank wear, the interaction between cutting speed and depth of cut was the third most significant factor (*p*-value = 1.2 × 10^−3^), then the interaction between feed rate and depth of cut (*p*-value = 1.6 × 10^−3^), followed by cutting speed (*p*-value = 3.1 × 10^−3^) and feed rate (*p*-value = 0.31).

It can be concluded that crater wear is accelerated more by machining a longer length than flank wear. This agrees with the results reported in [37], which concluded that, typically, the wear patterns formed when machining titanium alloys are craters generated by diffusion and adhesion. It was noted that depth of cut had a more significant effect than feed rate or cutting speed on both flank wear and crater wear. Nevertheless, crater wear is more affected than flank wear by changes in either cutting speed or feed rate. These results indicate the quality attainable when high-speed machining titanium alloys, because cutting speed has a relatively small effect, provided that the process parameters are optimized to avoid tool wear.

#### 3.2.2. Surface Roughness and High-Speed Machining of Ti6Al4V

**Figure 17:**
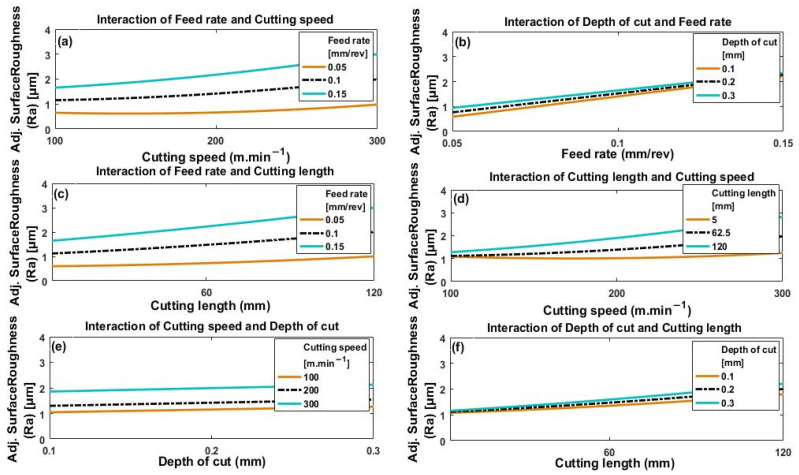
Plots depicting surface roughness (Ra) as a function of (**a**) cutting speed at different feed rates, (**b**) feed rate under different depths of cut, (**c**) cutting length at different feed rates, (**d**) cutting speed under different cutting lengths, (**e**) depth of cut for different cutting speeds, and (**f**) cutting length for different depths of cut.

Figure 17a–f shows how the interactions of the four process parameters (cutting speed, depth of cut, feed rate, and cutting length) impact on the value of Ra obtained. In Figure 17a we see that surface roughness increases slowly but monotonically with cutting speed, and that the greater the feed rate, the greater the resulting value of Ra. Thus, minimum roughness is obtained for minimum feed rate combined with minimum cutting speed. Surface roughness increases with increase in either cutting speed or feed rate. This is because of increased plastic deformation and build-up on the cutting edge, and increased friction and extrusion of the flank face of the tool [31].

Figure 17b shows a monotonic relationship between surface roughness and feed rate that appears linear. The deeper the depth of cut, the rougher the surface, but this effect was not substantial and decreased as feed rate increased. From Figure 17a,b it is observed that feed rate has a greater effect on the roughness of the surface than either depth of cut or if cutting speed of Ra increases.

In Figure 17c, cutting length and feed rate values are seen. Consequently, Ra is lower for short cutting lengths and lower feed rates, with substantial values of Ra at large values of cutting length and high feed rates. Thus, as mentioned above, the wear of the tool is accelerated by greater feed rates and increasing values of cutting length, this agrees with the results reported in [22]. It follows that surface roughness values for machined surfaces increase significantly with increase in feed rate and cutting length.

Figure 17d shows surface roughness as a function of cutting speed with changes in cutting length. It is seen that surface roughness is minimal at the lowest values of cutting speed and cutting length but increases with both cutting speed and cutting length to produce high levels of roughness. This is due to the increased tool wear that occurs at higher levels of cutting speed and cutting length.

Figure 17e shows that surface roughness increases only slightly with deeper cuts for all values of cutting speed, but a significant increase is noted with increase in cutting speed.

Figure 17f shows that surface roughness increases with cutting length for all values of depth of cut. It was also noted that depth of cut has a relatively significant effect at large values of cutting length. However, at smaller values of cutting length there was no noticeable effect on surface roughness due to changes in depth of cut.

**Figure 18:**
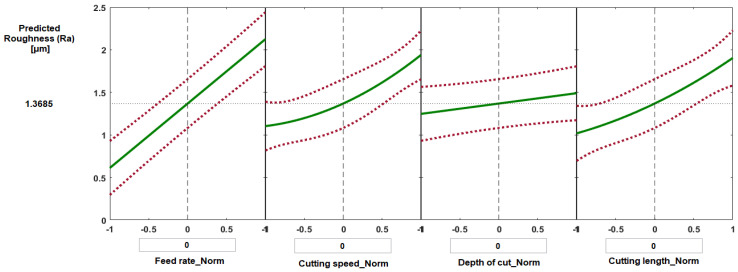
Prediction slice plots based on the developed regression model for surface roughness (Ra) of Ti6Al4V specimens.

Figure 18 shows selected plots for the prediction of (Ra) corresponding to cutting conditions at their average levels (feed rate 0.1 mm/rev, cutting speed 200 m·min^−1^, depth of cut 0.2 mm, and cutting length 62.5 mm). The predicted value of Ra was 1.368 μm. Table 4 shows the difference between experimental and predicted results for surface roughness. The range of error found to be from 2.5% to 5 % for randomly selected set of trials.

According to the ANOVA results, the process factor having the greatest impact on surface roughness was feed rate (*p*-value = 2.3 × 10^−13^) followed by cutting speed (*p*-value = 1.0 × 10^−5^) and cutting length (*p*-value = 1.7 × 10^−5^). Depth of cut had minimum effect on surface roughness (*p*-value = 8.5 × 10^−2^). The interaction of feed rate and cutting speed (*p*-value = 3.6 × 10^−3^) and the interaction of cutting length and cutting speed (*p*-value = 3.1 × 10^−3^) also had relatively little impact on surface roughness values.

The results show that turning at high cutting speed of 200 m/min using carbide inserts found to give approximately the same ranges of obtainable surface roughness and flank wear when compared with conventional machining at low cutting speed of 80 m/min, as seen in [38,39]. This also agrees with the results reported in [15], which led the authors to suggest machining Ti6Al4V alloys with high-speed machining for its high throughput when compared with conventional machining.

## 4. Conclusions

This study has presented a systematic investigation to assess the response of Ti6Al4V under high-speed machining. The results of previous tests [30,31] have been extended for a range of cutting conditions, and obtained values of flank, crater wear, and surface roughness have been examined and analyzed. The results have been modelled using a regression technique and analyzed using ANOVA to assess and better understand the physical phenomena involved and the effect of changes in process parameters, and their interactions, on wear and roughness.

The main conclusions are:

- The results showed that abrasion was the most important flank wear mechanism at high speed;
- Crater wear showed greater sensitivity to cutting length than did flank wear, confirming that typical tool wear when machining Ti6Al4V will be crater wear. In particular, crater wear increased by over 150% (on average), and flank wear increased by 40% (on average) when increasing cutting length from 40 to 120 mm;
- Rapid crater wear and flank wear were observed by increasing cutting speed from 100 to 200 m·min^−1^ while less effect was found by increasing cutting speed from 200 to 300 m·min^−1^. In particular, the average increase in crater wear and flank wear were 77% and 58%, respectively, when increasing cutting speed from 100 to 200 m·min^−1^, while 9% and 33% increase in crater wear and flank wear, respectively, were found by increasing cutting speed from 200 to 300 m·min^−1^;
- The combination between high cutting speeds (200 and 300 m·min^−1^) and low depth of cut resulted in obviously decreased flank wear;
- From ANOVA results, depth of cut and cutting length are found to have the greatest effects on both flank and crater wear;
- Flank wear was more affected by interactions between cutting conditions than crater wear, but was only slightly influenced by feed rate;
- Cutting length had a substantial effect on surface roughness which is explained as due to tool wear. In particular, surface roughness increased by 50% (on average) when increasing cutting length from 40 to 120 mm;
- ANOVA showed that during the high-speed machining of Ti6Al4V, the cutting speed has a relatively minor effect on tool wear and roughness of the surface compared to the other parameters, provided that the process parameters are optimized.

## Figures and Tables

**Table 1:** Cutting conditions used for experiments.

Cutting Condition	Levels
Feed rate (f) (mm/rev)	0.05, 0.15
Depth of cut (a_p_) (mm)	0.1, 0.3
Cutting speed (v_c_) (m·min^−1^)	100, 200, 300
Cutting length (l) (mm)	5, 40, 80, 120

**Table 2:** Validation results: measured vs. predicted crater wear.

#	Feed Rate (mm/rev)	Cutting Speed (m/min)	Depth of Cut (mm)	Cutting Length (mm)	Crater Wear (mm)		
					Measured	Predicted	Error %
1	0.15	200	0.3	120	0.375	0.36	4
2	0.05	100	0.1	80	0.091	0.0985	−8.2
3	0.05	300	0.1	40	0.108	0.109	1

**Table 3:** Validation results: measured vs. predicted flank wear.

#	Feed Rate (mm/rev)	Cutting Speed (m/min)	Depth of Cut (mm)	Cutting Length (mm)	Flank Wear (mm)		
					Measured	Predicted	Error %
1	0.15	200	0.3	120	0.774	0.719	7.1
2	0.05	30	0.3	40	0.653	0.58	11
3	0.15	100	0.1	80	0.218	0.255	−3.2

**Table 4:** Validation results: measured vs. predicted surface roughness.

#	Feed Rate (mm/rev)	Cutting Speed (m/min)	Depth of Cut (mm)	Cutting Length (mm)	Surface Roughness (µm)		
					Measured	Predicted	Error %
1	0.15	100	0.3	80	1.727	1.815	−5
2	0.15	200	0.3	40	1.891	1.94	2.5
3	0.05	300	0.1	120	1.335	1.299	2.6

## Data Availability

Not applicable.
